# Reduced audiovisual recalibration in the elderly

**DOI:** 10.3389/fnagi.2014.00226

**Published:** 2014-08-27

**Authors:** Yu Man Chan, Michael J. Pianta, Allison M. McKendrick

**Affiliations:** Department of Optometry and Vision Sciences, University of Melbourne, ParkvilleVIC, Australia

**Keywords:** audiovisual, multisensory, synchrony judgement, aging, adaptation

## Abstract

Perceived synchrony of visual and auditory signals can be altered by exposure to a stream of temporally offset stimulus pairs. Previous literature suggests that adapting to audiovisual temporal offsets is an important recalibration to correctly combine audiovisual stimuli into a single percept across a range of source distances. Healthy aging results in synchrony perception over a wider range of temporally offset visual and auditory signals, independent of age-related unisensory declines in vision and hearing sensitivities. However, the impact of aging on audiovisual recalibration is unknown. Audiovisual synchrony perception for sound-lead and sound-lag stimuli was measured for 15 younger (22–32 years old) and 15 older (64–74 years old) healthy adults using a method-of-constant-stimuli, after adapting to a stream of visual and auditory pairs. The adaptation pairs were either synchronous or asynchronous (sound-lag of 230 ms). The adaptation effect for each observer was computed as the shift in the mean of the individually fitted psychometric functions after adapting to asynchrony. Post-adaptation to synchrony, the younger and older observers had average window widths (±standard deviation) of 326 (±80) and 448 (±105) ms, respectively. There was no adaptation effect for sound-lead pairs. Both the younger and older observers, however, perceived more sound-lag pairs as synchronous. The magnitude of the adaptation effect in the older observers was not correlated with how often they saw the adapting sound-lag stimuli as asynchronous. Our finding demonstrates that audiovisual synchrony perception adapts less with advancing age.

## INTRODUCTION

It is important to correctly combine visual and auditory signals to obtain a coherent percept of events occurring in our surrounds. However, this is a non-trivial task due to the relative difference in the transmission speed of visual and auditory signals in air, and in the nervous system (King and Palmer, 1985). At a distance of around ten meters, the slower speed of sound in air is compensated by the faster speed of sound processing in the neural system, thus visual and auditory signals arrive at a common brain area at the same time. For source distances within ten meters, there is an increase in auditory-lead with decreasing source distance. Beyond ten meters, the amount of auditory-lag within an audiovisual signal increases with increasing source distance (e.g., lightning and thunder). Audiovisual processing needs to be adaptable to accommodate the different arrival times at different viewing/hearing distances. A common real world example is spectator sports (for example, tennis) where, when watching from the top of the stands, there is an asynchrony between the visual image of the racquet hitting the ball and the sound of the contact. This perceived audiovisual asynchrony is typically only noticeable for a brief period, and is no longer noticed as the game proceeds. The ability to adapt to crossmodal asynchrony is important for correctly relating events across different distances (Heron et al., 2007; Parsons et al., 2013).

Previous work using a range of different stimulus types has demonstrated such a shift in audiovisual synchrony perception after adapting to audiovisual asynchrony (Fujisaki et al., 2004; Vroomen et al., 2004; van Eijk et al., 2008; Vatakis et al., 2008). A classic study by Fujisaki et al. (2004) presented a continuous stream of asynchronous (auditory-lead or -lag) flash-click stimuli to young healthy participants for 3 min. The participants were then asked to judge if a subsequent audiovisual stimulus pair was synchronous or asynchronous. By measuring synchrony judgements across a range of stimulus onset asynchronies before and after the adaptation, the authors reported a shift in perceived synchrony in the direction of the adapted asynchrony. In other words, some stimuli that were perceived as asynchronous before adaptation were perceived as synchronous after the short-term adaptation. A similar shift in synchrony perception occurs for more complex and natural stimuli (Fujisaki et al., 2004; van Eijk et al., 2008; Vatakis et al., 2008; Asakawa et al., 2009, 2012; Tanaka et al., 2009, 2011).

Older people are more likely to perceive synchrony, or are more likely to have trouble separating temporally offset visual and auditory signals that are not relevant to each other (Hay-McCutcheon et al., 2009; DeLoss et al., 2013; Chan et al., 2014). We have recently shown that this observation cannot be entirely accounted for by an age-related reduction in unisensory detection thresholds (Chan et al., 2014). We scaled stimulus visual Gabor contrast and auditory sound pip intensity to individual detection thresholds, yet the older adults still had wider audiovisual synchrony windows (average width of 224 ms) compared to the younger group (average width of 166 ms). Our findings indicate that age-related differences in the ability to separate auditory and visual signals in time are not due to peripheral visual or hearing decline. A decrease in the ability to perceive asynchrony may predict a reduction in audiovisual asynchrony adaptation.

Besides synchrony judgements, other methods used to assess audiovisual temporal perception include the sound induced flash illusion, as well as temporal order judgements. For the former, audiovisual interaction is quantified as the susceptibility to the illusion. For the latter, participants are required to judge whether the visual or the auditory signal is presented first within an audiovisual pair. Previous studies have shown that both younger and older people are equally susceptible to the sound induced flash illusion when the flash and sound are presented 70 ms apart. However, the older group experienced the sound induced flash illusion more often than the younger group when the flash and sound signals are separated by 270 ms (Setti et al., 2011a,b). This finding has been interpreted to indicate an increased audiovisual interaction resulting in difficulty in separating temporally offset visual and auditory signals with age, consistent with reports for audiovisual synchrony judgements (Hay-McCutcheon et al., 2009; Chan et al., 2014). However, in a temporal order judgment task, Fiacconi et al. (2013) failed to find the same age effect. Love et al. (2013) and van Eijk et al. (2008) have compared the results from audiovisual synchrony judgment and temporal order judgment tasks and suggest that the two tasks tap into different underlying neural mechanisms for temporal perception (van Eijk et al., 2008; Love et al., 2013). Audiovisual synchrony judgment gives a more accurate measure of the perception of subjective simultaneity, whereas temporal order judgment provides a better measure of the smallest audiovisual asynchrony detectable by the perceptual system (van Eijk et al., 2008).

Our study was designed to test whether healthy older individual exhibit altered adaptation to audiovisual asynchrony. After adapting older and younger observers to sound-lag asynchrony, both groups showed an expansion of their synchrony window in the direction of the adapted asynchrony, but the degree of expansion was smaller for the older group. However, in contrast to predictions, the reduced expansion in the older group could not be accounted for by the perceived synchrony of the adapting stimuli.

## MATERIALS AND METHODS

### PARTICIPANTS

Fifteen younger (22–32 years old) and 15 older (64–74 years old) adults participated in the experiment. Younger participants were recruited from the University of Melbourne, and older adults were recruited from the university and the community via advertisements posted in community newspapers. Inclusion criteria included having normal or corrected-to-normal vision of 6/7.5 or better, and normal hearing for age. Hearing was assessed in a quiet laboratory space using an audiometer with headphones (Garson Stadler GSI 18 audiometer, Eden Prairie, MN, USA). Normal hearing was defined as having audiometric thresholds less than 35 decibels hearing level (dB HL) at 4 kHz, and less than 25 dB HL at all other tested frequencies (0.25, 0.5, 1, and 2 kHz), according to the International Organization for Standardization (ISO) standard on hearing by age and gender (ISO 7029:2000 Acoustics). The study was approved by the Human Research Ethics Committee of University of Melbourne and informed consent was obtained from all participants in accordance with the Declaration of Helsinki.

### EQUIPMENT

The experiment was controlled by software written in MATLAB 7.6.0 (R2008a; Mathworks, Boston, MA, USA) and run on a personal computer (Dell Precision T3500, Round Rock, TX, USA). The visual stimulus was presented using a ViSaGe (Cambridge Research Systems, Cambridge, UK) to drive a cathode ray tube monitor (Sony Trinitron Multiscan G520 – mean luminance: 100 cd/m^2^, frame rate: 100 Hz, 1024 × 768 pixels, Tokyo, Japan) that was gamma corrected on a weekly basis. Responses were collected using a CB6 response box. The ViSaGe also initiated sound presentation through a set of headphones (Sennheiser HD 205, Wedemark, Germany), by triggering a multifunction processor [Tucker-Davis Technologies (TDT) RX6, Alachua, FL, USA] that drove a programmable attenuator (TDT PA5) and a headphone driver (TDT HB7). Timing precision was verified prior to starting on the main experiment using an oscilloscope. Participants stabilized their head position by resting on a chin rest positioned 100 cm from the monitor.

### STIMULI

**FIGURE 1:**
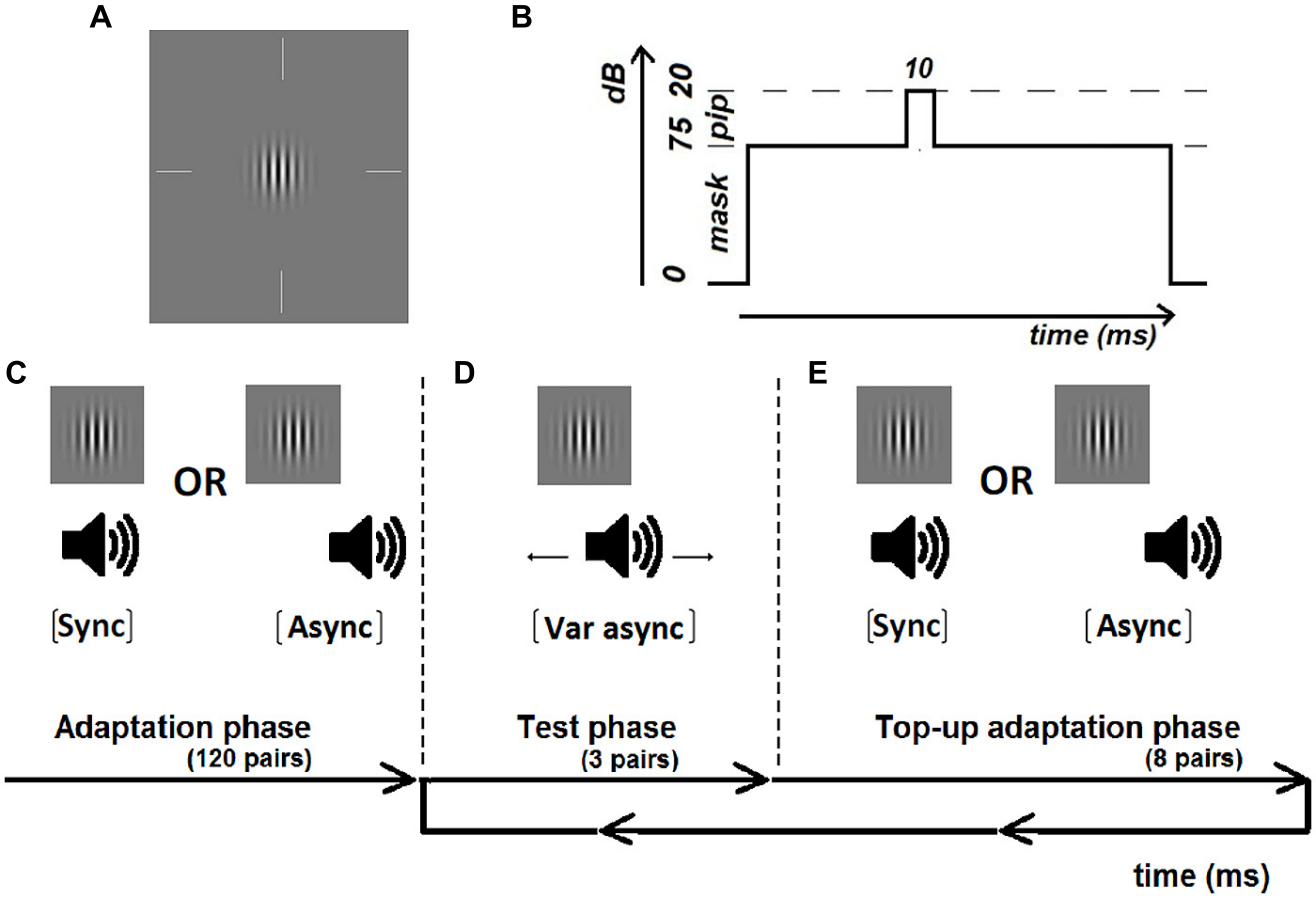
**Illustration of the visual Gabor (A) and auditory masked tone pip (B) and the experimental procedure including an initial adaptation period of 120 adaptation pairs (C) either synchronous or asynchronous (sound-lag asynchrony 230 ms), test periods of 3 pairs with variable asynchrony (D) and top-up adaptation periods of eight pairs (E)**.

The visual stimulus was a vertically striped Gabor of 3 c/deg (85% contrast), with the standard deviation of the Gaussian envelope defined as the reciprocal of the spatial frequency (= 0.33^∘^), that was presented for one monitor frame (frame rate: 100 Hz) (**Figure 1A**). The auditory stimulus was a pure tone pip (20 dB, 10 ms duration; 2.5 ms onset and offset ramp) of 500 Hz presented binaurally through headphones over a pure tone mask (75 dB, 1.5 s duration, 100 ms onset and offset ramp) of the same frequency (**Figure 1B**). The onset of the tone pip was jittered between 200 and 300 ms from the auditory mask onset. Stimulus onset asynchrony was defined as the time difference between the onset timings of the tone pip and the Gabor. Adaptation pairs were either synchronous or asynchronous with a fixed sound-lag asynchrony of 230 ms. By comparing the shift in synchrony perception to each observer’s perception after adapting to synchrony, we reduced the amount of inter-subject variability which can result from individual differences in prior experience (Navarra et al., 2010; Alm and Behne, 2013).

### PROCEDURE

Each test run consisted of an initial adaptation phase, followed by a repeated looping of a test phase and a top-up adaptation phase until all test trials were completed. In the initial adaptation phase, participants were exposed to 120 adaptation pairs that were either synchronous or asynchronous (**Figure 1C**). The adaptation pairs were separated by a duration randomly chosen from a uniform distribution from 1000 to 1100 ms. In order to maintain attention during the adaptation phase, 20 randomly occurring catch trials were interleaved where the visual Gabor contained horizontal instead of vertical stripes. Participants were instructed to press on a button if the orientation of the Gabor was horizontal. All participants responded correctly to all of the catch trials. The adaptation phase was approximately 3 min in duration.

At the end of the adaptation phase, participants judged the synchrony for three test pairs (**Figure 1D**) before being re-exposed to eight top-up adaptation pairs (**Figure 1E**). Participants indicated whether the test stimuli appeared synchronous or asynchronous via a button press. No feedback was given to the participants regarding the likelihood of encountering synchronous or asynchronous pairs. Responses were self-paced, with the next test-pair presented 500 ms after the button press. Individual synchrony windows were measured across eleven asynchronies using a method-of-constant-stimuli (MOCS). These asynchronies were manually adjusted by the researcher (YMC) for each individual to span from approximately 100% synchronous response to 0% synchronous response. Based on previous work in the lab, a test range of ±450 and ±550 ms was sufficient to reach asymptotic response (i.e., 100 and 0% proportion of synchronous responses) for younger and older participants, respectively. Therefore each younger participant began with a practice run with MOCS steps of ±450, ±330, ±190, ±100, ±50, and 0 ms. If asymptotic response was not achieved with the test range used in the practice run, the test range for the actual test runs was extended to ±500, ±400, ±300, ±200, ±100, 0 ms. Each older participant began with a practice run with MOCS steps of ±550, ±450, ±350, ±250, ±100, and 0ms. If asymptotic response was not achieved with this test range, the MOCS steps used in the actual test runs were changed to ±600, ±450, ±350, ±250, ±100, and 0 ms. Asymptotic response was obtained in all of the actual test runs for all participants. Within each run, test pairs were tested in sets of three until each of the eleven asynchrony steps were tested for four repeats. The initial adaptation pairs and top up adaptation pairs in each test run were always identical, either synchronous or asynchronous. Each adaptation condition was tested for four repeats (total of 16 repeats at each asynchrony step). The order of the eight test runs was randomized within and between participants to avoid order effects. Each run, including the adaptation phase and test and top-up adaptation phases, lasted for no more than 10 min. Total test time to complete the two adaptation conditions lasted around 80 min. Participants were given breaks (no fixed duration) whenever required. Consequently each observer participated in ∼180 min in total of testing including initial screening, practice runs and breaks.

### ANALYSIS

**FIGURE 2:**
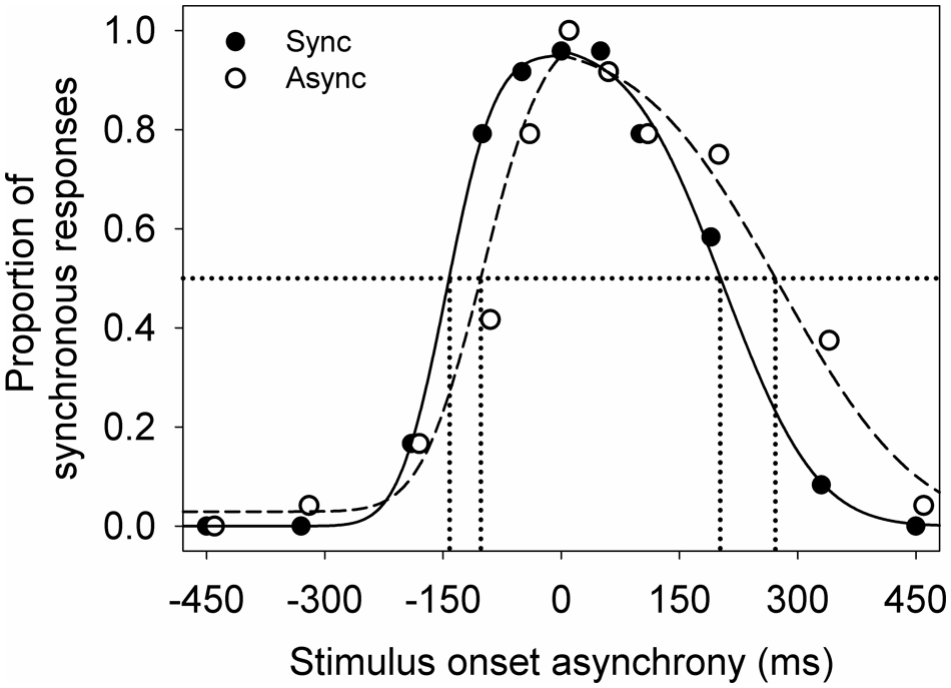
**An example of the audiovisual synchrony window for a young participant after adapting to synchrony (filled) and to asynchrony (unfilled).** Lines indicate the Gaussian normal distributions fitted using maximum likelihood estimation (sync: solid; async: dashed). The symbols are jittered slightly along the *x*-axis for illustrative purposes.

Each participant’s data was averaged across the four test repeats for each adapting condition. Then two independent cumulative Gaussian distributions were fitted to the averaged data using least sum of squares (**Figure 2**). One cumulative Gaussian distribution was fit to the data from earliest sound-lead asynchrony tested to 0 ms, and the second cumulative Gaussian was fit to the data from 0 ms to the latest sound-lag asynchrony tested. The cumulative Gaussian distribution was defined by

(1) f(t)=FP+(1−FP−FN)×[G(t,μ,σ)]⁢

where *G*(*t*, μ, σ) was the cumulative Gaussian distribution with mean (μ) and standard deviation (σ) for stimulus asynchrony value *t*. FP and FN represented the proportions of false positive and false negative responses, respectively, (i.e., the asymptotic error values). The means (μ) of the fitted distributions gave sound-lead and sound-lag synchrony thresholds (i.e., the sound-lead asynchrony that was perceived as synchronous 50% of the time, and the sound-lag that was perceived as synchronous 50% of the time). The standard deviations (σ) defined the participant’s asynchrony discrimination for sound-lead and sound-lag pairs. The width of the audiovisual synchrony window was calculated as the difference between the sound-lead and sound-lag thresholds. The magnitude of the adaptation effect was quantified as the difference between these parameters (sound-lead threshold, sound-lag threshold, asynchrony discrimination sensitivities and window width) for the two adapting conditions. The curve fit aided in the data analysis but was not intended to have any physiological meaning. The root mean squared error of the individually fitted psychometric functions fell below 0.183 for the younger cohort and below 0.179 for the older group. We used a repeated-measures analysis of variance (RM-ANOVA) to test for effects of age or adapted condition on the sound-lead synchrony threshold, sound-lag synchrony threshold, and window width estimates.

## RESULTS

**Figure 2** shows an example of the audiovisual synchrony windows obtained from a young observer. There is a high proportion of synchronous response when the visual and auditory signals were presented at synchrony (0 ms on the *x*-axis) and at a small stimulus onset asynchrony. The proportion of synchronous responses decreases with increasing asynchrony (moving leftward and rightward along the *x*-axis). This observer showed a smaller shift in sound-lead threshold than sound-lag threshold after adapting to asynchrony (unfilled circles, dashed line).

**FIGURE 3:**
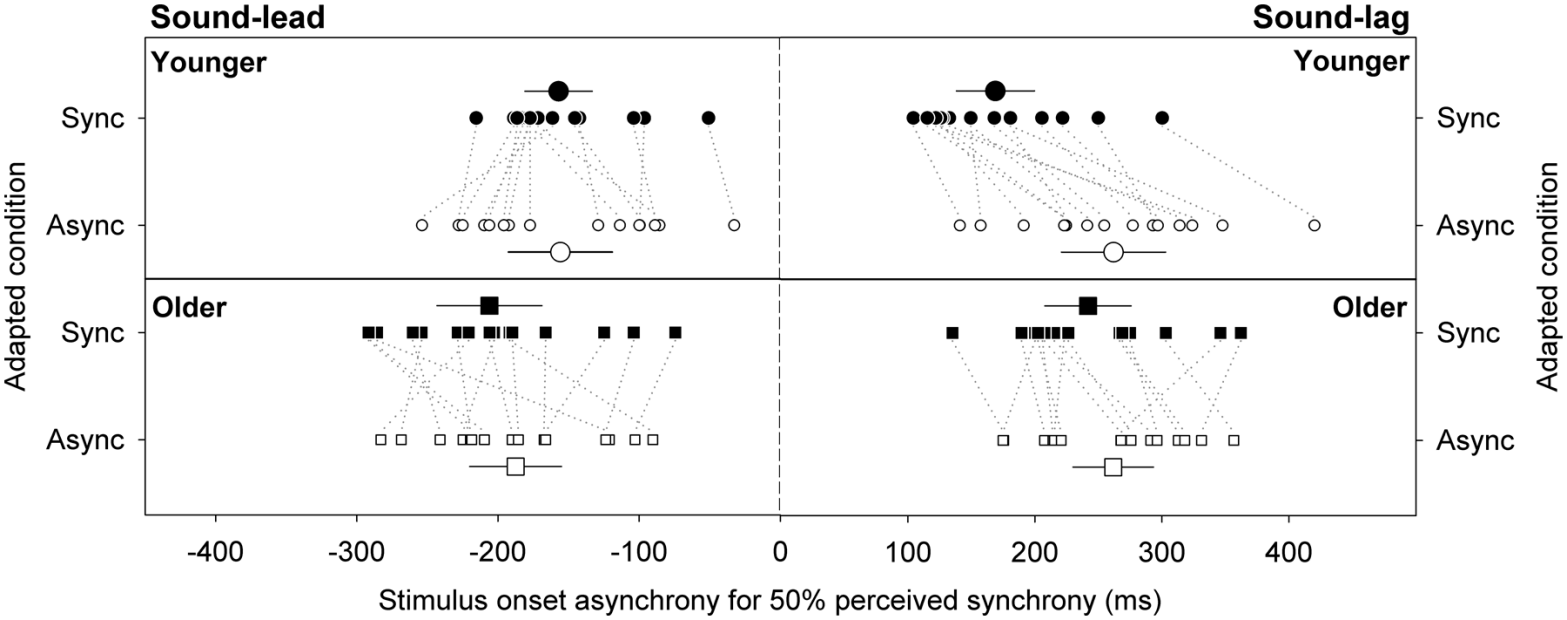
**Stimulus onset asynchrony for perceiving sound-lead and sound-lag stimuli as synchronous 50% of the time.** Large symbols represent the group averages for the younger (circles) and older (squares) cohorts. Error bars are 95% confidence intervals. Smaller symbols represent individual data, with dotted lines connecting the data for each individual, to emphasize the direction and magnitude of the shift (closed symbols: adaptation to synchrony, open symbols: adaptation to sound-lag asynchrony).

**Figure 3** illustrates the synchrony thresholds for sound-lead and sound-lag stimuli for the two adapting conditions. In a mixed design repeated measures ANOVA that compared between the two adapting conditions, two threshold types and two age groups, there was a main effect of threshold type [*F*(1,28) = 31.82, *p* < 0.001), adapting condition [*F*(1,28) = 12.43, *p* = 0.001) and age [*F*(1,28) = 4.76, *p* = 0.04) on the synchrony thresholds. The main effect of threshold type was not dependent on the age group (no significant interaction between threshold type and age group: *F*(1,28) = 0.04, *p* = 0.84). The significantly different synchrony thresholds between the adapting conditions was, however, dependent on age group (significant interaction between adapting condition and age group: *F*(1,28) = 11.74, *p* = 0.002) and on threshold type (significant interaction between adapting condition, threshold type and age group: *F*(1,28) = 4.53, *p* = 0.04). No other statistics were significant.

### SYNCHRONY THRESHOLDS FOR SOUND-LEAD STIMULI

Previous reports showed that adaptation to sound-lag asynchrony expanded the audiovisual synchrony window asymmetrically toward greater sound-lag asynchrony (Fujisaki et al., 2004). We examined the effect of adaptation on the sound-lead and sound-lag thresholds independently in two separate ANOVAs. The left panels in **Figure 3** plot the sound-lead thresholds for the two adapting conditions. There was a main effect of age on sound-lead threshold [*F*(1,28) = 4.27, *p* = 0.048) where older observers on average required greater sound-lead asynchrony to perceive asynchrony (adapted to synchrony: -206, adapted to asynchrony: -187 ms) than the average younger group (adapted to synchrony: -157, adapted to asynchrony: -156 ms). There was no effect of adaptation on sound-lead thresholds for both age groups [no main effect of adapting condition: *F*(1,28) = 1.05, *p* = 0.31]. No other statistics were significant.

### SYNCHRONY THRESHOLDS FOR SOUND-LAG STIMULI

The right panels of **Figure 3** show the sound-lag thresholds. Adaptation to sound-lag shifts mean sound-lag thresholds toward greater asynchronies (i.e., in the direction of the adapted asynchrony) for both age groups [main effect of adapting condition: *F*(1,28) = 39.96, *p* < 0.001], however, the group average shift in the young observers (adapted to synchrony: 169 ms, adapted to asynchrony: 262 ms; shift: +93 ms) is greater than the group average shift in the older cohort [adapted to synchrony: 242 ms, adapted to asynchrony: 262 ms; shift: +20 ms; significant interaction between age group and adapting condition: *F*(1,28) = 16.78, *p* < 0.001]. No other statistics were significant.

**FIGURE 4:**
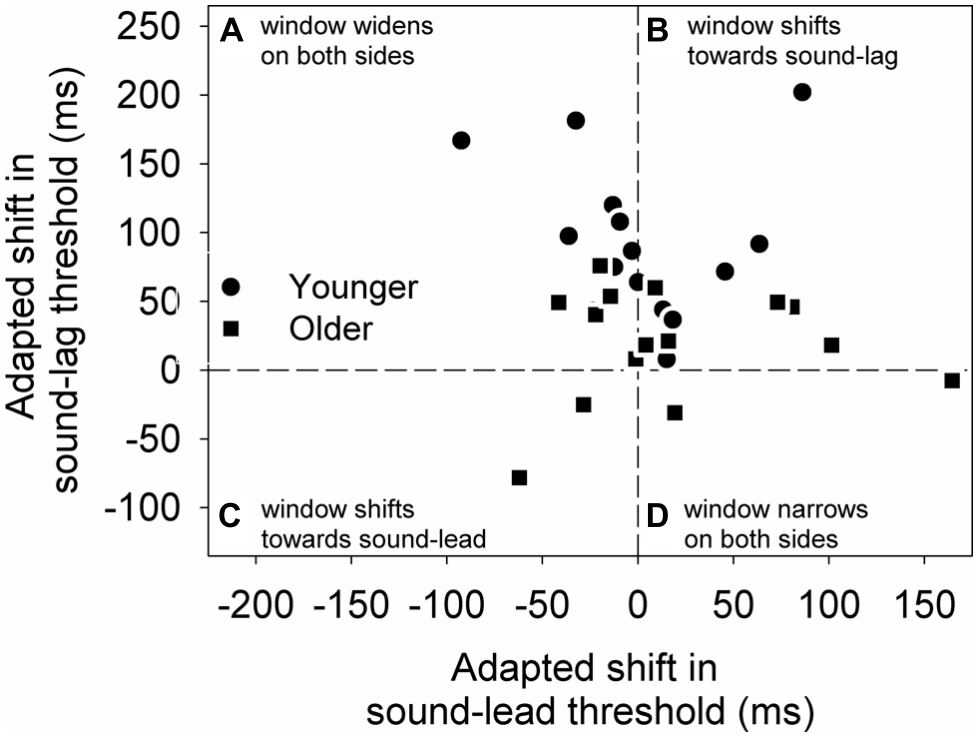
**The adapted shift (async-sync) in sound-lag thresholds is plotted against the adapted shift in sound-lead threshold for each older (squares) and younger (circles) participant.** This graph is segmented into four quadrants to illustrate the individuals who, after adapting to asynchrony, showed a widening on both sides of the synchrony window **(A)**, a shift of the entire window toward sound-lag **(B)**, toward sound-lead **(C)**, and a narrowing on both sides of the window **(D)**.

To better illustrate the change in the overall synchrony window post-adapting to asynchrony, we calculated the difference between the two adapted conditions (adapted shift in threshold) for each individual’s sound-lead and sound-lag thresholds (**Figure 4**). For both age groups, most data points appear above zero on the *y*-axis, meaning the majority of our participants either showed a widening on both sides of their synchrony window (**Figure 4A**) or a shift of the entire window toward sound-lag (**Figure 4B**).

### AUDIOVISUAL SYNCHRONY WINDOW WIDTHS

For both adapting conditions, synchrony windows were on average wider in the older (448, 449 ms after adaptation to synchrony and asynchrony, respectively) than the younger (326, 418 ms) group. After adapting to asynchrony, the synchrony window widened for the younger group (an increase of 92 ms), but the change was small for the older group (an increase of 1 ms).

### AUDIOVISUAL ASYNCHRONY DISCRIMINATION SENSITIVITY

The standard deviation of the fitted cumulative Gaussians provided an estimate of each participant’s asynchrony discrimination sensitivity for sound-lead and sound-lag pairs. After adapting to synchrony, the group averaged standard deviation for the younger group was 36 ms (95% confidence interval: ±10 ms) for sound-lead pairs and was 48 ms (±11 ms) for sound-lag pairs. The group averaged standard deviation for the older adults was 41 ms (±13 ms) for sound-lead pairs, and 57 ms (±20 ms) for sound-lag pairs. After adapting to asynchrony, the group averaged standard deviations for the younger cohort were 37 ms (±16 ms) and 57 ms (±21 ms) for sound-lead and sound-lag pairs, respectively, and in the older cohort were 61 ms (±19 ms) and 65 ms (±18 ms). These estimates were analyzed in a mixed design ANOVA that compared between the two adapted conditions, two age groups and two synchrony threshold types (2 × 2 × 2). There was no main effect of age [*F*(1,28) = 2.04, *p* = 0.16], nor of adaptation condition [*F*(1,28) = 2.70, *p* = 0.11], and no significant interaction effects (all *p*> 0.05).

### IS THE ADAPTATION EFFECT RELATED TO HOW THE ADAPTOR WAS PERCEIVED?

**FIGURE 5:**
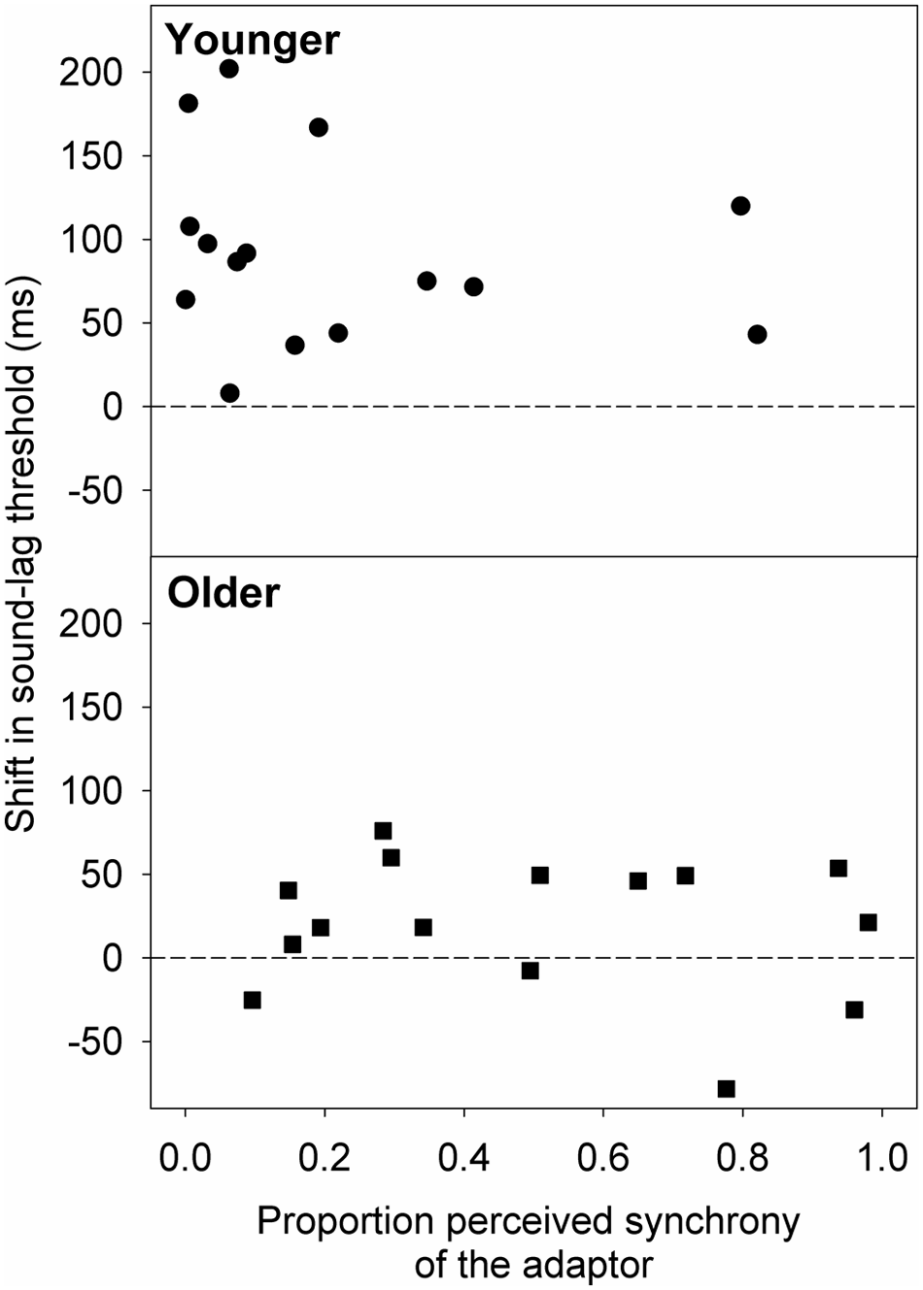
**Shift in the sound-lag threshold for each older (squares) and younger (circles) observer, relative to their proportion perceived synchrony for the adaptor (230 ms sound-lag) at baseline (i.e., after adapting to synchrony).** A positive shift indicates a shift in the direction of the adapted asynchrony.

We extended our analysis to see if the smaller magnitude of adaptation in the older group was due to the adaptor appearing synchronous more often to the older than the younger observers. Therefore, we used the best-fit psychometric functions obtained after adaptation to synchrony to estimate the proportion of perceived synchrony for the adaptor asynchrony (sound-lag asynchrony of 230 ms). We then conducted linear regression analyses on the relationship between the shift in sound-lag threshold and the proportion of perceived synchrony for the adaptor asynchrony (**Figure 5**). No statistically significant linear dependence of the adaptation shift magnitude on perceived synchrony of the adaptor was detected for either age group [younger: slope (±95% CI) = -38.26 (-159.51, 82.99); *t*(13) = -0.68, *p* = 0.51; older: slope = -20.73 (-98.51, 56.70); *t*(13) = -0.58, *p* = 0.57].

### IS THE ADAPTATION EFFECT RELATED TO INDIVIDUAL SYNCHRONY WINDOW WIDTHS?

**FIGURE 6:**
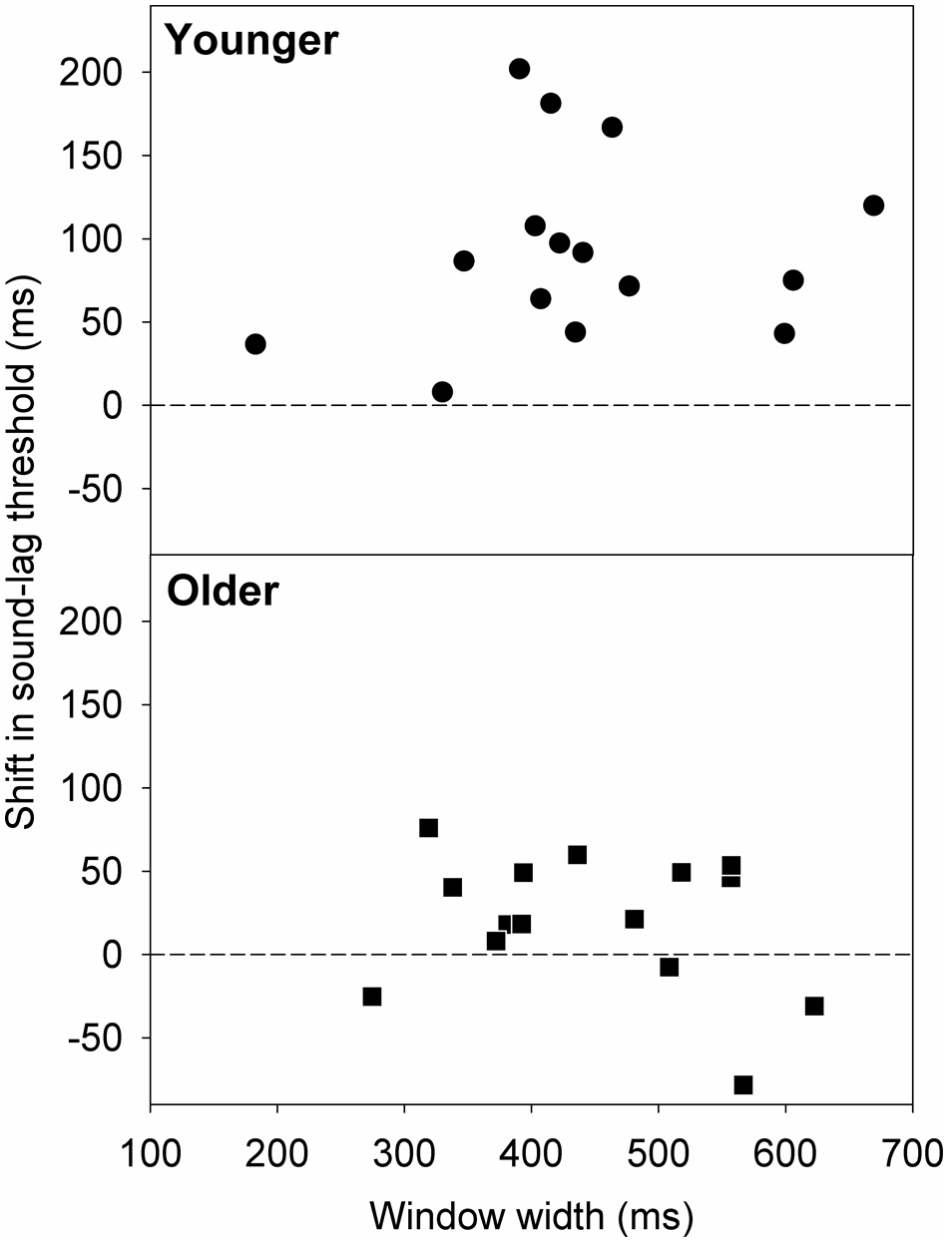
**Shift in the sound-lag threshold for each older (squares) and younger (circles) observer, relative to their synchrony window width at baseline (i.e., after adapting to synchrony).** A positive shift indicates a shift in the direction of the adapted asynchrony.

Van der Burg et al. (2013) found that rapid asynchrony adaptation to the audiovisual pair presented immediately before the test pair was dependent on individual synchrony window width. Participants who had wider synchrony windows exhibited a larger magnitude of adaptation effect (Van der Burg et al., 2013). We investigated if this trend applies similarly to our data. **Figure 6** plots the shift in the sound-lag threshold as a function of window width. A simple linear regression analysis showed no statistically significant linear dependence of the adaptation shift magnitude on synchrony window width [younger: slope = 0.10; 95% confidence interval: -0.31, 0.51; *t*(13) = 0.54, *p* = 0.60; older: slope = -0.11; 95% confidence interval: -0.33, 0.12; *t*(13) = -1.00, *p* = 0.33].

## DISCUSSION

Our results show an age-related widening of the audiovisual synchrony time window, consistent with recent reports on aging in synchrony perception (Hay-McCutcheon et al., 2009; Chan et al., 2014) and in the audiovisual sound-induced illusion (DeLoss et al., 2013). We also demonstrate that, with healthy ageing, elderly observers recalibrate their sound-lag threshold to a lesser extent when they are exposed to the same asynchrony adaptation as younger adults. The degree to which the adaptor is perceived as asynchronous does not influence the size of the shift in perceived sound-lag synchrony in either age group, so the smaller adaptation effect seen in the elderly observers is unlikely to be due to differences in how the adaptor was perceived by the young and older cohorts. Although rapid adaptation to asynchronous pairs was dependent on synchrony window width (Van der Burg et al., 2013), our study on short term adaptation showed no significant relationship between adaptation effect and window width.

We did not explicitly measure cognitive performance of our participant, however, it is unlikely that differences in cognitive capacity or differential task difficulty could have resulted in the age effect found. Our older participants were recruited from the community, were fit and active, and passed general screening of vision and hearing to ensure no significant age-related sensory organ damage. There were four current or retired university staff. The rest were active elderly citizens who were still involved in casual paid work and volunteer work in the community. Recent aging study on the audiovisual sound-induced illusion also showed an unlikely role of selective attention on the increased audiovisual integration (increased illusion) with age (DeLoss et al., 2013). Our older participants were active elderly citizens (64–74 years) recruited from the university and the community. All passed the inclusion criteria of having healthy vision and hearing that are normal for their age. As expected for individuals of this age, hearing thresholds were less than 35 decibels hearing level (dB HL) at 4 kHz, and less than 25 dB HL at all other tested frequencies (0.25, 0.5, 1, and 2 kHz; ISO 7029:2000 Acoustics). Data collected during the testing was clean, as demonstrated by all participants reaching 100% synchronous responses when the audiovisual stimulus was at physical synchrony, and 0% synchronous responses when the visual and auditory signals were separated by at most 600 ms. There was also no difference in the spread of the psychometric functions between groups hence the ability to make asynchrony discrimination judgements was similar between the two groups. Our data provides no evidence for either differential levels of task learning, fatigue, or attention. Selective attention has been previously shown to be an unlikely explanation for age-related increase in the audiovisual sound-induced flash illusion (more likely to combine the beeps with the flash; DeLoss et al., 2013). All participants were trained with practice trials to ensure they were confident with the task and were performing it correctly before proceeding onto the main experiment. It is also worth noting that previous studies have shown that procedural and perceptual learning in older adults is similar to that of younger adults for visual tasks (McKendrick and Battista, 2013), and is not simply an effect of task practice but rather a change in the underlying neural process (Andersen et al., 2010).

After adaptation to synchronous audiovisual stimuli, elderly observers have a wider synchrony window compared to young observers. This is consistent with previous studies on the effect of aging on audiovisual synchrony judgment without adaptation (Hay-McCutcheon et al., 2009; Chan et al., 2014). By measuring asynchrony-synchrony judgements for simple flash-pip stimuli in a two-interval-forced-choice design, previous data from our laboratory measured wider synchrony windows in the older observers (465 ms; 61–72 years, mean age of 66 years; seven males) than in younger observers (319 ms; 21–32 years, mean age of 25 years; five males). This widening of the window was independent of response criteria bias and age-related decline in visual contrast sensitivity and hearing thresholds, since data were collected using stimuli scaled to visual and auditory sensitivity for each individual. The width estimates in our younger (326 ms) and older (448 ms) cohorts in the current study are comparable to those we have measured previously.

Adaptation to sound-lag produces a change in the synchrony window of young observers. Consistent with previous data (Fujisaki et al., 2004; Vroomen et al., 2004; Navarra et al., 2009, 2012), there was no significant change to sound-lead thresholds, whereas sound-lag thresholds increased, thus resulting in an asymmetric widening of the window. The elderly observers showed a similar pattern of results, but the magnitude of the adaptation-induced shift was reduced. This reduced recalibration in the elderly observers was not related to how frequently the adaptation stimuli were perceived as synchronous or asynchronous (**Figure 5**) or synchrony window width (**Figure 6**). It is, however, possible that the trend for a greater variance in perceived synchrony of the adaptor in the older group could have contributed to the lack of a significant relationship between the perceived synchrony of the adaptor and adaptation effect. On the other hand, Van der Burg et al. (2013) reported a direct relationship between rapid asynchrony adaptation effect and synchrony window width in their young adults. The absence of this relationship in our data on short term adaptation can be argued as the recruitment of different neural mechanism for rapid and short term audiovisual asynchrony adaptation. Rapid adaptation is more likely to be an early sensory effect, whereas the short term adaptation in our study alters later higher level neural processes (Van der Burg et al., 2013). It is, however, worth noting that such rapid recalibration may possibly influence our findings as the data was collected in test triplets. There is insufficient data to analyse test order effects within the triplets directly.

After adapting to asynchrony, sound-lag thresholds shifted to an average of 262 ms for both age groups. One possible explanation for our results is that 262 ms may be the average optimal position for the sound-lag threshold when the audiovisual system is exposed to an adaptor with a sound-lag asynchrony of 230 ms. Consequently, the reduced response in the elderly may simply reflect the fact that the average sound-lag end of their synchrony window is closer to this limit prior to adaptation. Such an explanation would predict that those individuals closer to this optimal point would demonstrate less adaptation than those further away. However, the individual data, as plotted in **Figure 3**, are not readily consistent with this suggestion.

The smaller adaptation with age could be explained by the need for longer adaptation duration in the older group to reach the same amount of adaptation effect as the younger group. In a visuomotor experiment that compared the motor response before and after visual prism adaptation, older people required longer adaptation duration to the prism before they were able to correctly point to the visual target (Fernández-Ruiz et al., 2000). However, this is a purely vision-based adaptation that is possibly confined to the neural processes responsible for visual processing only. We do not know if the same explanation of longer adaptation duration with age can be directly applied to the multisensory context of our study.

The computational approach and neural basis by which the brain encodes temporal information regarding auditory and visual stimuli is poorly understood. One proposed mechanism is that unisensory neural processing speed is altered by the adaptation process (Di Luca et al., 2009; Navarra et al., 2009). It has been argued that such a model predicts a uniform recalibration, independent of the specific test stimulus asynchronies that may be presented post-adaptation (Di Luca et al., 2009; Navarra et al., 2009). Roach et al. (2011) demonstrate that the magnitude of recalibration is non-uniform, but instead varies according to the timing offsets between the auditory and visual pairs. The pattern of human behavior that they observed was fit by a computational population-coding model of audiovisual timing tuned neurons. The observed variable bias in recalibration as a function of audiovisual timing offset was well captured by a model that incorporated reduced response gain in the neurons tuned to the adapted asynchrony (Roach et al., 2011).

The neural and anatomical locus of such a population of audiovisual timing tuned neurons, and whether they are functionally or structurally affected by aging is not known. Neurons with such temporal specificity may be located in multisensory brain areas like the superior colliculus, as shown in single cell recording in cats (Meredith et al., 1987). In aged primates, there is evidence for the tuning properties of visual neurons to become less selective with advancing age, for example broader direction tuning and orientation tuning in the primary visual cortex of cats (Hua et al., 2006) and primates (Schmolesky et al., 2000). A proposed mechanism is a reduction of inhibition which is supported by the presence of fewer GABAergic neurons in the cat visual striate cortex (Hua et al., 2008), and by experiments demonstrating that orientation tuning can be regained by administration of inhibitory GABA agonists (Leventhal et al., 2003). To our knowledge, similar neurophysiological data is not available for multisensory areas. Broadening of neuronal tuning properties may be a generalized feature of aging in sensory cortices, and it is also possible that the number of neurons contributing to a population of cells encoding audiovisual timing might also reduce with age. Future planned experiments should be able to collect data suitable for specific application of a population-coding model similar to that described in Roach et al. (2011). Comparison of age groups using such strategies may enable insight regarding whether differences in the patterns of behavior between older and younger adults are consistent with either (or both) a broadening of neuronal tuning or drop-out of cells. Electrophysiological experiments comparing evoked potentials between age groups before and after adaptation to asynchrony might also provide evidence for or against the alternate suggestion of an altered neural latency mechanism (Di Luca et al., 2009; Navarra et al., 2009).

In conclusion, our findings demonstrate that the recalibration response in older people differs from that of younger adults. For the stimulus conditions used in our experiments, older adults demonstrated reduced recalibration to the prevailing audiovisual timing environment, which was not related to their baseline percept of the adapting asynchrony. The specific neural basis for this difference, and how it impacts on sensory performance in more naturalistic environments, warrants further study.

## Conflict of Interest Statement

The authors declare that the research was conducted in the absence of any commercial or financial relationships that could be construed as a potential conflict of interest.
